# Matriptase-Induced Phosphorylation of MET is Significantly Associated with Poor Prognosis in Invasive Bladder Cancer; an Immunohistochemical Analysis

**DOI:** 10.3390/ijms19123708

**Published:** 2018-11-22

**Authors:** Koji Yamasaki, Shoichiro Mukai, Takahiro Nagai, Kozue Nakahara, Masato Fujii, Naoki Terada, Akinobu Ohno, Yuichiro Sato, Yoshinobu Toda, Hiroaki Kataoka, Toshiyuki Kamoto

**Affiliations:** 1 Department of Urology, Faculty of Medicine, University of Miyazaki, Miyazaki 889-1692, Japan; koji_yamasaki@med.miyazaki-u.ac.jp (K.Y.); takahiro_nagai@med.miyazaki-u.ac.jp (T.N.); kozue_nakahara@med.miyazaki-u.ac.jp (K.N.); masato_fujii@med.miyazaki-u.ac.jp (M.F.); naoki_terada@med.miyazaki-u.ac.jp (N.T.); tkampro@med.miyazaki-u.ac.jp (T.K.); 2 Section of Pathology, Faculty of Medicine, University of Miyazaki, Miyazaki 889-1692, Japan; akkun@med.miyazaki-u.ac.jp; 3 Section of Diagnostic Pathology, Faculty of Medicine, University of Miyazaki, Miyazaki 889-1692, Japan; yuichiro_sato@med.miyazaki-u.ac.jp; 4 Department of Clinical Laboratory Science, Tenri Health Care University, Nara 632-0018, Japan; todayadot@gmail.com; 5 Oncopathology and Regenerative Biology Section, Faculty of Medicine, University of Miyazaki, Miyazaki 889-1692, Japan; mejina@med.miyazaki-u.ac.jp

**Keywords:** matriptase, hepatocyte growth factor, MET, bladder cancer

## Abstract

Hepatocyte growth factor (HGF) plays an important role in cancer progression via phosphorylation of MET (*c-met* proto-oncogene product, receptor of HGF). HGF-zymogen (pro-HGF) must be processed for activation by HGF activators including matriptase, which is a type II transmembrane serine protease and the most efficient activator. The enzymatic activity is tightly regulated by HGF activator inhibitors (HAIs). Dysregulated pro-HGF activation (with upregulated MET phosphorylation) is reported to promote cancer progression in various cancers. We retrospectively analyzed the expression of matriptase, phosphorylated-MET (phospho-MET) and HAI-1 in tumor specimens obtained from patients with invasive bladder cancer by immunohistochemistry. High expression of phospho-MET and increased expression of matriptase were significantly associated with poor prognosis, and high matriptase/low HAI-1 expression showed poorer prognosis. Furthermore, high expression of matriptase tended to correlate with phosphorylation of MET. Increased expression of matriptase may induce the ligand-dependent activation of MET, which leads to poor prognosis in patients with invasive bladder cancer.

## 1. Introduction

Bladder cancer is the world’s 9th most common malignancy, with 357,000 cases reported in 2002 [1]. Histopathologically, 90% of bladder cancers are urothelial carcinoma, 5% are squamous cell carcinoma, and less than 2% are adenocarcinoma or other variants [2]. Approximately 75% of patients present with non-muscle-invasive bladder cancer (NMIBC), which is effectively managed by transurethral resection of bladder tumor (TURBT) [3]. On the other hand, radical cystectomy and urinary diversion is generally considered for patients with muscle-invasive bladder cancer (MIBC). Neoadjuvant chemotherapy may be considered as an option to improve the cure rate [4]. However, MIBC remains lethal. Cancer-specific survivals at 5 years for pT2 and pT3 have been reported as 65–78% and 31–55%, respectively, [5,6] and the development of more effective treatment is essential.

HGF is a multifunctional growth factor reported to have an important role in tumor progression through its specific receptor tyrosine kinase MET, the c-met proto-oncogene product [7]. HGF is primarily secreted by fibroblasts as an inactive single-chain precursor (pro-HGF) that lacks biological activity and requires proteolytic cleavage for activation into its two-chain mature form [8,9]. Matriptase, which is a Type II transmembrane serine protease (TTSP), is the most efficient pericellular activator of pro-HGF. The binding of HGF to MET activates tyrosine kinase activity, which in turn results in autophosphorylation of tyrosine residues in the activation loop, and generates multi-docking sites for molecules which mediate downstream signal transduction [10]. In cancer tissue, activation of HGF/MET signaling axis occurs mainly by paracrine fashion, and several cancers express HGF, which leads to autocrine-loop-style MET activation [8,11]. The activation of the HGF/MET signaling axis is reported to be involved in tumor progression, including increased cell-proliferation, motility, invasiveness (epithelial-mesenchymal transition: EMT) and anti-apoptotic potential [8,11]. In bladder cancer, phosphorylation of MET is reported to correlate with the progression and poor prognosis [12].

Matriptase is reported to be expressed in various epithelial cells and to have an essential role in the maintenance of epithelial barrier formation in skin and intestine [8]. On the other hand, increased expression of matriptase is observed in many cancers, including breast, ovarian, uterine, prostate, colon, cervical, skin, renal, pancreatic, esophageal, head and neck carcinomas [13,14,15,16,17,18,19,20,21]. In these cancers, matriptase has a significant correlation with increased invasive and metastatic activity through its potential to activate several growth factors, including pro-HGF, protease-activated receptor 2 (PAR-2), pro-platelet-derived growth factor D (PDGF-D) and pro-macrophage stimulating protein (MSP)-1 [8]. In physiological condition, enzymatic activity is tightly regulated by HGF activator inhibitor (HAI)-1. Expression of matriptase in urothelial cancer cells has not been evaluated, and its function is not well analyzed. This is the first study to analyze the expression of matriptase and HAI-1, and to examine correlation with the phosphorylation of MET in bladder cancer specimens by immunohistochemistry.

## 2. Results

### 2.1. Immunohistochemical Study

#### 2.1.1. Expression of MET, Phospho-MET, Matriptase and HAI-1 in Cancer Tissue

**Figure 1:**
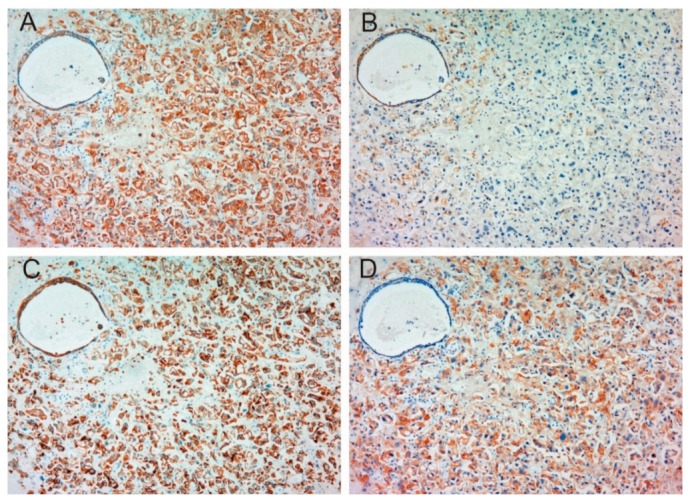
Immunohistochemical appearance of total MET (**A**), phospho-MET (**B**), matriptase (**C**) and HAI-1 (**D**) in serial tissue section of high-grade urothelial carcinoma (patient 10, ×200 magnification). Increased expression of total MET is observed (**A**), whereas phosphorylation is downregulated (**B**). Although matriptase is expressed in the majority of cancer cells (**C**), the activity may be regulated by HAI-1 (**D**). This case shows a representative pattern of HAI-1-induced downregulation of MET phosphorylation.

**Figure 2:**
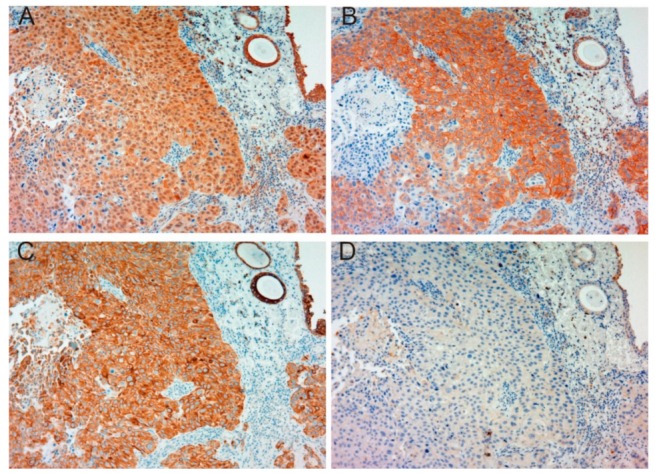
Immunohistochemical appearance of total MET (**A**), phospho-MET (**B**), matriptase (**C**) and HAI-1 (**D**) in serial tissue sections of high-grade urothelial carcinoma (patient 26, ×200 magnification). Increased expression of total MET (**A**) and upregulation of phosphorylated MET (**B**) are observed. Expression of matriptase is also increased (**C**). Dysregulation of matriptase may be promoted by downregulation of HAI-1 (**D**). This case shows a representative pattern of HAI-1 downregulation-induced upregulation of MET phosphorylation.

**Figure 3:**
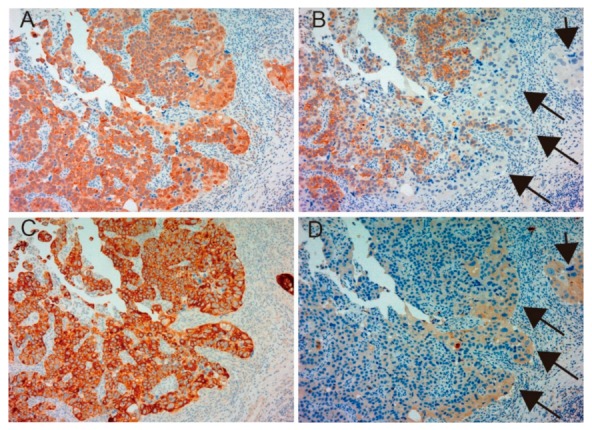
Immunohistochemical appearance of total MET (**A**), phospho-MET (**B**), matriptase (**C**) and HAI-1 (**D**) in serial tissue sections of high-grade urothelial carcinoma (patient 23, ×200 magnification). Dominant expression of total MET (**A**) and matriptase (**C**) is observed. Phosphorylation of MET is also upregulated (**B**); however, downregulation is observed in part (arrows). In contrast, increased expression of HAI-1 is observed in the area with downregulated phospho-MET (**D**, allows). This case shows a representative pattern of reciprocal expression of HAI-1 and phospho-MET.

**Figure 4:**
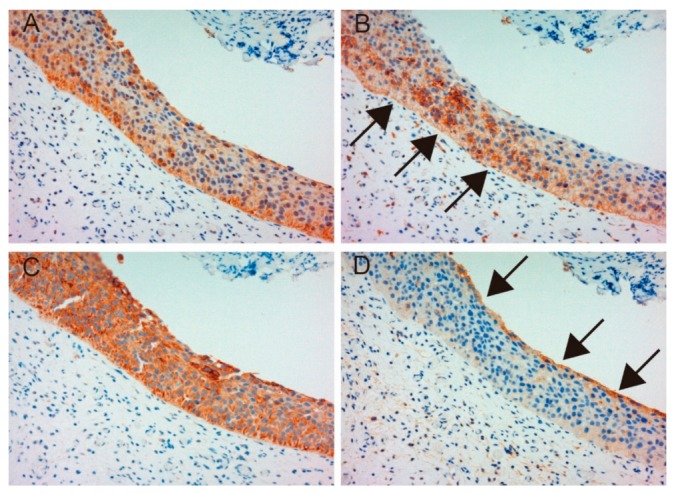
Immunohistochemical appearance of total MET (**A**), phospho-MET (**B**), matriptase (**C**) and HAI-1 (**D**) in serial tissue sections of normal urothelium (patient 23, ×200 magnification). Total MET and matriptase are expressed in the majority of normal urothelial cells (**A**,**C**). However, phosphorylation of MET is upregulated in the basal area of the urothelium (**B**, arrows), and HAI-1 is expressed in umbrella cells (**D**). These patterns are representative of non-malignant urothelium adjacent to a cancer lesion.

Table 1 shows patient characteristics. Immunohistochemical appearance is shown in Figure 1, Figure 2, Figure 3 and Figure 4. Positive staining of all molecules, which was defined as membranous staining with or without cytoplasmic stain, was observed in cancer cells. As shown in Table 2, MET, phosphorylated MET (phospho-MET), matriptase and HAI-1 were highly expressed in 12 cases (46%), 7 cases (27%), 18 cases (69%) and 11 cases (42%), respectively. Although increased expression of total MET was observed in patients 10–18, phosphorylation was downregulated. High expression of HAI-1 was also observed in these patients, suggesting HAI-1-induced regulation of MET phosphorylation (Figure 1). In contrast, upregulated MET phosphorylation was observed in patients 24–26, whereas HAI-1 was downregulated (Figure 2). Considering these results, HAI-1 may have an important role in the regulation of ligand-dependent MET activation in bladder cancer. On the other hand, both expression of HAI-1 and phosphorylation of MET were upregulated in patients 20–23. Histopathological findings of these cases are shown in Figure 3. Of interest, phosphorylation of MET and expression of HAI-1 were observed reciprocally in cancer tissues. These findings also suggest the significant role of ligand-dependent activation of MET and HAI-1-induced regulation in bladder cancer. In peri-cancerous normal urothelium, total MET and matriptase expressed in the majority of urothelial cells, whereas expression of HAI-1 was observed in umbrella cells and phosphorylation of MET was enhanced in the basal area of the urothelium (Figure 4)

#### 2.1.2. Correlation between Pathological T Stage and Expression of Each Molecule

We analyzed the correlation between pathological T stage and the result of immunohistochemistry (Table 3). High expression of MET and phospho-MET was significantly associated with higher T stage (*p* = 0.027 and *p* = 0.03 respectively). However, no significant correlation was observed between T stage and expression of matriptase and HAI-1.

#### 2.1.3. Correlation between Matriptase, HAI-1 and Phospho-MET

Next, we analyzed the correlation between each molecule (Table 4). Although correlation between HAI-1 and phospho-MET failed to achieve statistical significance, high expression of matriptase tended to correlate with the phosphorylation of MET (*p* = 0.068).

#### 2.1.4. Cancer-Specific Survival According to the Expression of Each Molecule

**Figure 5:**
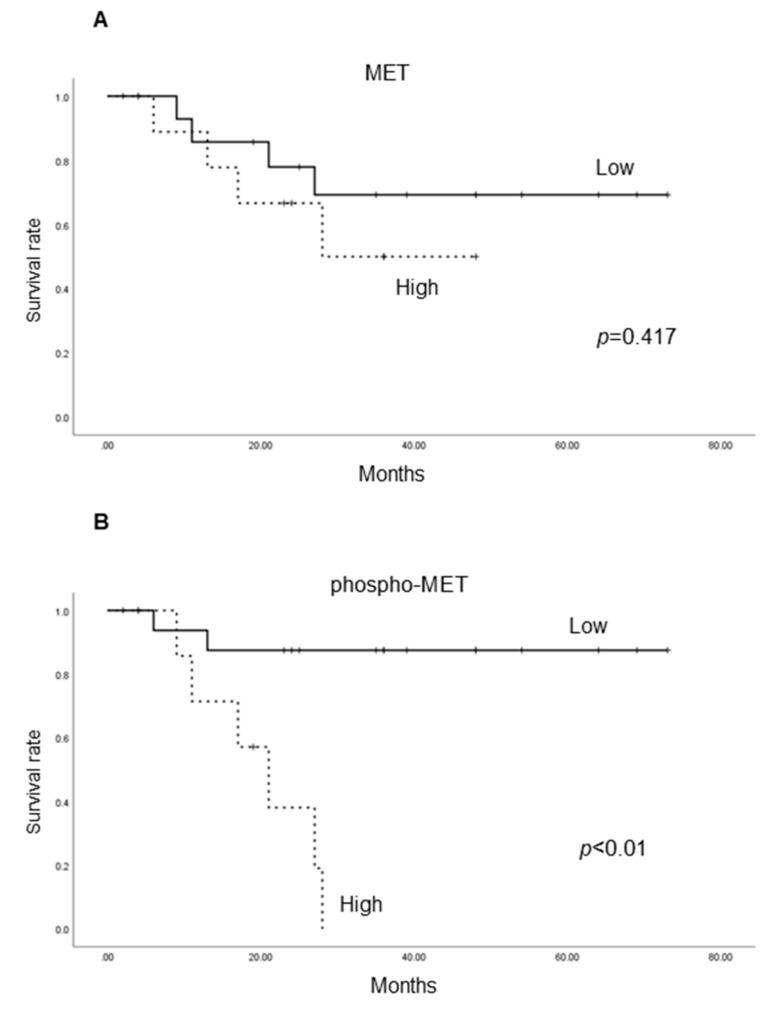

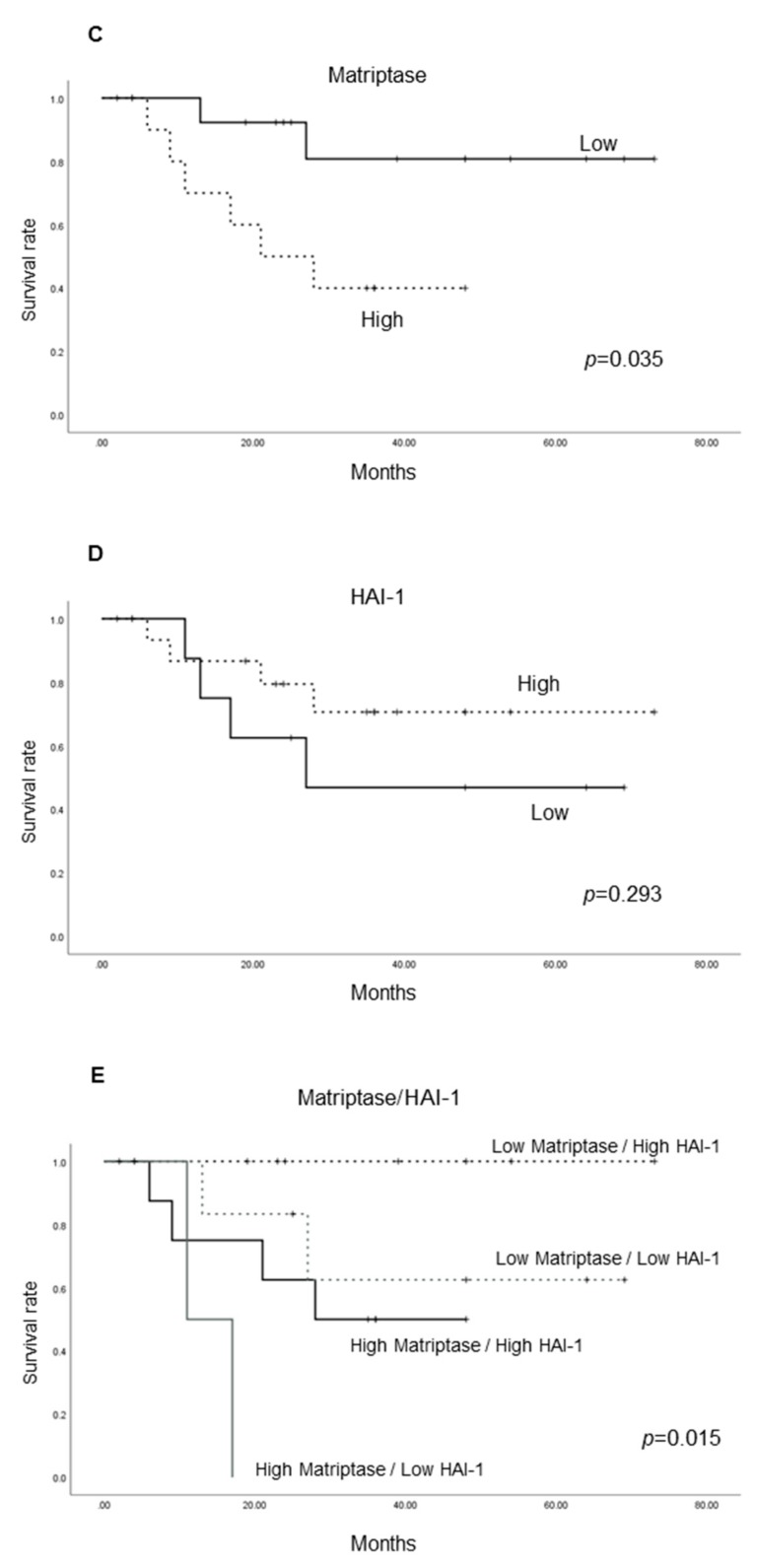
Comparison of cancer-specific survival rates (CSS) of high-grade urothelial carcinoma patients in total MET (**A**), phospho-MET (**B**), matriptase (**C**) and HAI-1 (**D**) and matriptase in combination with HAI-1 (E). Survival distributions were compared by log-rank statistics. Expression of total MET and HAI-1 is not significantly associated with CSS. However, expression of phospho-MET and matriptase is significantly associated with CSS. Analysis of subgroup is shown (**E**). The low matriptase/ high HAI-1 group reveals favorable prognosis, whereas poor prognosis is observed in the high matriptase/ low HAI-1 group.

We analyzed cancer-specific survival according to the expression of each molecule (Figure 5). High expression of phospho-MET and matriptase was both significantly correlated with poor prognosis. Although high expression of MET tended to correlate with poor prognosis, statistical significance was not achieved. Then, in response to reports that an imbalance of matriptase and HAI-1 was associated with tumor aggressiveness [22], we evaluated cancer-specific survival in subgroup (Figure 5E). The low matriptase/ high HAI-1 expression group showed favorable prognosis, whereas the high matriptase/ low HAI-1 expression group showed a significantly poorer prognosis.

## 3. Discussion

MET is a receptor tyrosine kinase which consists of an extracellular alpha chain and a single-pass transmembrane β chain showing an architecture of disulfide-linked heterodimer [23,24]. The intracellular segment comprises a juxtamembrane domain responsible for the downregulation of MET kinase activity, an activation loop that actuates MET kinase activity after Tyr1234 and Tyr1235 phosphorylation, and a carboxy-terminal multifunctional docking site comprising Tyr1349 and Tyr1356, which leads to downstream signaling [23,24,25]. MET phosphorylation leads to the activation of several major intracellular signaling pathways that eventually promote cell proliferation, survival (anti-apoptosis), motility, invasiveness, drug resistance, the maintenance of the cancer stem cell-like phenotype, and epithelial-mesenchymal transition (EMT) in several cancer cells [23,24]. High expression of MET with worsening prognosis is reported in a great number and variety of human cancers [18,23,24,26].

Three means of MET activation are observed in human cancers. Activating point mutations in hereditary papillary renal cell carcinoma is a widely known self-activation system [23]. Overexpression of MET leads to oligomerization, which causes reciprocal ligand-independent activation [24]. In addition, HGF-dependent activation in autocrine or paracrine system is also a major activation system of MET [18,25,26]. *MET* amplification is a mechanism of resistance to epidermal growth factor receptor-targeted tyrosine kinase inhibitors (EGFR-TKIs) in lung cancer [27]. In contrast to this, overexpression of HGF is seen in a significant number of lung cancer patients with EGFR-TKIs-resistance overlapping *MET* amplification [27]. In addition, HGF expression is increased significantly in patients with acquired EGFR-TKIs-resistance [27]. Examination of resistance to anti-MET therapy also revealed the importance of HGF-promoted resistance in drug potency and efficacy [28]. Furthermore, phosphorylation of MET with auto-activating mutation was enhanced to a significant degree by HGF stimulation [29]. Therefore, activation of pro-HGF in the MET signaling axis is of significant importance in cancer progression [27,28,29], providing a rationale for targeting pro-HGF activation systems in anti-MET treatment. Indeed, high expression levels of pro-HGF are frequently observed in various human cancers, particularly in cancer-associated stromal cells.

Matriptase gene (*ST14*) is located on human chromosome 11q24-25 [30]. The gene encodes 855 amino acids, and the molecular weight of the protein is 80–90-kDa [31]. Matriptase is a Type II transmembrane serine protease (TTSP), which is composed of a single pass hydrophobic transmembrane domain, a short intracellular amino terminus (N-terminus) domain and a larger extracellular carboxy-terminal serine protease domain [30]. This protease is synthesized as an inactive, single-chain zymogen, and activation requires two sequential endoproteolytic cleavages [30]. Matriptase was first discovered in breast cancer cell line T-47D, and subsequently purified from human milk [32]. Matriptase was highly expressed in breast cancer cell lines and involved in cancer progression through activation of the HGF/MET signaling axis [33,34]. Co-expression of matriptase and MET was also reported to correlate with poor prognosis in head and neck cancer, and renal cell carcinoma [18,35]. In bladder cancer, although phosphorylation of MET (Y1349) was reported to correlate with high T stage, metastasis and poor prognosis, expression of matriptase has not been evaluated [36]. Our study revealed that upregulated phosphorylation of MET and expression of matriptase significantly correlated with poor prognosis, and high expression of matriptase with downregulated HAI-1 tended to correlate with worse prognosis in patients with invasive bladder cancer. To the best to our knowledge, this is the first report analyzing matriptase and HAI-1 expression in bladder cancer.

HAI-1 is known as a pericellular inhibitor of pro-HGF-activating proteases [37]. HAI-1 is encoded by the *SPINT1* gene located on human chromosome 15q15.1 [8]. HAI-1 was initially purified from conditioned medium of human gastric cancer cell (MKN45), and is characterized by two functional Kunitz-type inhibitor domains [37]. HAI-1 can efficiently inhibit the proteolytic activity of HGF activator, matriptase, prostasin, hepsin, transmembrane protease, serine (TMPRSS) 13, human airway trypsin-like protease, kallikrein 1-related peptidase (KLK) 4 and KLK5 due to Kunitz-type inhibitor domain binding [8,37]. It has been reported that HAI-1 is expressed in various epithelial cells, and possible suppressive role in cancer progression [8]. Reduced HAI-1 expression reported to be associated with poor prognosis in patients with prostatic, breast, ovarian and endometrial cancer [38,39,40,41,42]. Interestingly, in our investigation, patients with high expression of matriptase and low expression of HAI-1 had significantly poorer prognosis compared with patient with the opposite expression despite the absence of correlation between HAI-1 expression and cancer-specific survival. However, further examination is needed to clarify whether HAI-1 is an efficient suppressor of bladder cancer.

## 4. Materials and Methods

This was a retrospective study using clinical data from clinical records and tumor specimens obtained from paraffin-embedded blocks. The experimental protocol was approved by the Ethical Review Committee of Miyazaki University (O-0132, 6/3/2017). We examined a series of 26 specimens of bladder cancer collected by radical cystectomy at our hospital between 2010 and 2014. The bladder cancers were staged according to TNM classification, and pathological diagnosis was performed by two pathologists in accordance with the World Health Organization (WHO) classification of tumors.

### 4.1. Immunohistochemistry and Analysis

We prepared formalin-fixed paraffin-embedded sections according to the standard procedures. Specimens were used for staining and immunohistochemistry. Anti-human MET (total MET) rabbit polyclonal antibody and anti-human MET (Tyr1235, phosphorylated, phospho-MET) were purchased from Immuno-Biological Laboratories (Gunma, Japan) and anti-human ST14/matriptase polyclonal antibody was from LifeSpan Biosciences (Seattle, WA, USA). Anti-HAI-1 antibody was from R&D systems (Minneapolis, MN, USA). Immunohistochemistry was conducted by processing sections for antigen retrieval (microwaved in 10 mM citrate buffer, pH 6.0 for 10 min), followed by treatment with 3% H_2_O_2_ in methanol for 10 min and washing in phosphate-buffered saline (PBS) twice. After blocking in 3% bovine serum albumin and 5% goat serum in phosphate buffered saline for 2 h at room temperature, sections were incubated with primary antibodies overnight at 4 °C. Negative controls excluded the primary antibody. Sections were washed in PBS and incubated with EnVision-labeled polymer reagent (DAKO, Carpinteria, CA, USA) for 30 min at room temperature. Sections were exposed with nickel, cobalt-3, 3-diaminobenzidine (Immunopure Metal Enhanced DAB Substrate Kit; Piece, Rockford, IL, USA), and counterstained with hematoxylin. The phospho-MET immunostaining procedure has been described previously [25].

Immunoreaction staining intensity was judged by percentage of bladder cancer cells in which the cancer cell membranes were stained with or without staining of cytoplasm (e.g., if 80 out of 100 cells were stained, staining was 80%): staining of >80%, strongly positive (2+); 20–80%, positive (1+); 5–20%, weakly positive (±); <5%, negative (−). Evaluation was performed by two experienced pathologists. We regarded a 2+ finding as high expression, and 1+, ± and – findings as low expression for all molecules.

### 4.2. Statistical Analysis

SPSS statics, version 25.0 (SPSS, Chicago, IL, USA) was used to assess statistical parameters. To analyze follow-up data, the Kaplan-Meier method was used to calculate cancer-specific survival, and survival distributions were compared by log-rank test. χ^2^-test was used to determine associations, and a *P*-value of less than 0.05 was set for statistical significance.

## 5. Conclusions

Upregulated phosphorylation of MET and expression of matriptase were significantly associated with poor prognosis in patients with invasive bladder cancer. Immunohistochemical findings showed reciprocal expression of HAI-1 and phospho-MET, which revealed the importance of HAI-1-induced regulation of MET phosphorylation as a major synergic role in the progression of bladder cancer. In addition, it was indicated that increased expression of matriptase may induce the ligand-dependent activation of MET.

## Figures and Tables

**Table 1:** Patient characteristics.

Age (median)		73
Gender	Male	14
	Female	12
Neoadjuvant chemotherapy	+	12
	-	14
pT stage	≤ T1	6
	T2	15
	≥ T3	5
Follow-up period (months, median)		26

pT stage: pathological T stage.

**Table 2:** Comparative immunoreactivity of MET, phospho-MET, HAI-1 and matriptase.

Patient	MET	p-MET	HAI-1	Matriptase
1	H	L	L	L
2	L	L	L	L
3	L	L	L	L
4	L	L	L	L
5	L	L	L	L
6	L	L	H	L
7	L	L	H	L
8	L	L	H	H
9	L	L	H	L
10	H	L	H	H
11	H	L	H	L
12	H	L	H	L
13	H	L	H	L
14	H	L	H	H
15	H	L	H	H
16	H	L	H	L
17	H	L	H	H
18	H	L	H	H
19	L	L	H	L
20	L	H	H	L
21	L	H	H	H
22	L	H	H	H
23	H	H	H	H
24	L	H	L	L
25	L	H	L	H
26	H	H	L	H

H: high expression, L: low expression. p-MET: phosphorylation of MET, HAI-1: hepatocyte growth factor activator inhibitor type-1.

**Table 3:** Correlation between pathological T stage and expression of each molecule.

Molecules		pT Stage			
		1	2	≥3	*p* Value
MET	Low	4	10	0	0.027
	High	2	5	5	
Phospho-MET	Low	6	8	5	0.030
	High	0	7	0	
Matriptase	Low	2	5	1	0.845
	High	4	10	4	
HAI-1	Low	4	9	2	0.647
	High	2	6	3	

Low: low expression, High: high expression. Significance was determined by χ^2^ test.

**Table 4:** Correlation between matriptase, HAI-1 and phospho-MET.

		Matriptase			HAI-1		
		Low	High	*p* Value	Low	High	*p* Value
Phospho-MET	Low	13	2	0.068	5	14	0.418
	High	6	5		3	4	

Significance was determined by χ^2^ test.

## Abbreviations

HGF	Hepatocyto Growth Factor
HAI	Hepatocyto Growth Factor Activator Inhibitor
RNA	Rebonucleic Acid
NMIBC	Non-Muscle Invasive Bladder Cancer
MIBC	Muscle Invasive Bladder Cancer
TURBT	Transurethral Resection of Bladder Tumor
TTSP	Type II Transmembrane Serine Protease
EMT	Epithelial-Mesenchymal Transition
PAR	Protease Activated Receptor
PDGF	Platelet-Derived Growth Factor
MSP	Macrophage Stimulating Protein
NC	Negative Control
EGFR	Epidermal Growth Factor Receptor
TKI	Tyrosine Kinase Inhibitor
TMPRSS	Transmembrane Protease, Serine
KLK	Kallikrein 1-Related Peptidase
DNA	Deoxyribonucleic Acid
